# Augmented Reality as a Resource for Improving Learning in the Physical Education Classroom

**DOI:** 10.3390/ijerph17103637

**Published:** 2020-05-21

**Authors:** Antonio-José Moreno-Guerrero, Santiago Alonso García, Magdalena Ramos Navas-Parejo, María Natalia Campos-Soto, Gerardo Gómez García

**Affiliations:** Department of Didactics and School Organization, University of Granada, 51001 Ceuta, Spain; ajmoreno@ugr.es (A.-J.M.-G.); salonsog@ugr.es (S.A.G.); magdalena@ugr.es (M.R.N.-P.); ncampos@ugr.es (M.N.C.-S.)

**Keywords:** augmented reality, physical education, teaching innovation, classroom climate, motivation

## Abstract

Recently, there has been a proliferation of technopedagogical practices, based on the application of active teaching and learning processes through the use of information and communication technologies (ICT). The main objective of this work is to analyse the impact of training action through the use of augmented reality in physical education for the development and acquisition of spatial orientation, as opposed to more traditional training based on the exhibition method. The methodology developed is quantitative, through a quasi-experimental design post-test in 140 high school students in the field of physical education. The results indicate that all of the dimensions show a very high, significant relationship. The greatest difference in average is observed in motivation. In contrast, the smallest difference, although significant, is observed in the grades given by the teachers themselves. It can be concluded that the method of teaching through augmented reality is effective in teaching high school students in the subject of physical education, especially for the acquisition of spatially oriented content.

## 1. Introduction

Nowadays, we live in a society in which the use of information and communication technologies (ICT) has acquired a relevant value in all social sectors [1,2] especially in the field of health [3] and education [4,5].

With regard to the latter, the importance of ICTs as a resource to encourage student participation and to motivate and activate interest in their learning process should be highlighted [6]. 

Along these lines [7], they maintain that, currently, the educational practices carried out in most educational centres are not adapted to the context in which students work outside of school hours, causing a lack of motivation and interest in them. In view of the above, it would be advisable to put into practice new teaching methodologies [8] that include the use of technology as a pedagogical resource [9] and to be able to offer updated training in accordance with the reality of the 21st century student [10,11]. There is no doubt that we are facing a digital society [12], which is why one of the priority goals of the current education system should be to equip classrooms with technological supplies that guarantee the use of ICTs in the teaching and learning processes [13]. Among the different methodologies that favour the improvement of these processes, active methodologies [14] stand out, characterised by the active role of the student. In this case, teaching does not revolve around the teacher or the content of the subject, but rather around the students and the tasks that are put into practice to promote learning [15].

Within these methodologies, it is important to highlight augmented reality (AR), which in recent years has been of great importance in the educational context, as demonstrated by numerous experiences that have been put into practice [16,17]. AR applied to university students promotes understanding, reflection [18], creativity, participation and innovation, but above all, motivation [19], since it allows for a more complete perception and interaction with the real world through complementary information provided by a technological device [20], favouring teaching and learning processes and the progress of ubiquity in educational centres [21]. 

### 1.1. Augmented Reality in Teaching and Learning Processes

AR is one of the emerging digital technologies that has aroused the most interest in the educational field [22], because it allows interaction between real and virtual space [23]. This is the fundamental characteristic which defines this type of technology and which differentiates it from virtual reality: the coexistence of both spaces simultaneously [24], which makes it possible to enrich the physical environment by means of the superimposition of virtual information which can appear in different formats and which can be easily activated using mobile devices in some cases [25,26,27].

The AR has different stages that increase the complexity of the effect they produce and require the use of more sophisticated devices as they increase: level 0 refers to hyperlinks that are generated by QR codes and can be easily activated from a mobile phone, level 1 uses markers, level 2 requires the use of a GPS locator, level 3 manages to add virtual elements to the real space through special glasses and level 4 requires the use of thermal fingerprints and contact lenses [28].

This technopedagogical tool used in education allows for an endless number of possibilities that favour significant learning by students through the approach of contexts to the classroom with which they can interact, as well as visualisation [29,30].

The key to the success of AR lies in the number of advantages it brings to education [31]. Numerous authors have shown that it significantly increases student motivation, since it allows the content of study subjects to be presented in an attractive and novel way, awakening interest and curiosity in the teaching process [32,33,34], which is a fundamental prelude to achieving significant learning [35].

Methodological trends in education focus on increasing motivation to the highest possible level [36], since other key consequences for education arise from this increase, such as increased involvement and interest in the subject [37]. 

Experts have also shown that AR contributes to the promotion of student leadership through experimentation with materials and content in an autonomous manner and from a constructivist approach, produced through discovery learning [38], which is the most effective type of learning, according to specialists [39]. It also facilitates cooperative and collaborative work, which are other aspects pursued by the current style of education that bets on learning among equals [40].

Another potential of AR is its adaptation to different educational stages [41]. It is a very versatile tool that can be adapted to very different student profiles, making it possible to use it effectively from Pre-school to Higher Education [42]. 

In addition to all of these factors, AR contributes to education by improving digital competencies and bringing virtual content into the classroom, which allows students to contextualise their learning and facilitate understanding of different subjects [43,44]. The final result is an improvement in academic performance and the classroom climate [45,46].

### 1.2. Augmented Reality in Physical Education

The AR is used as a teaching methodology in different branches of education [47]. This scope covers very different fields, such as science engineering [48], business [49], health [50] and linguistics [51], among many others. It is also used in tourism, leisure, marketing and transport [52]. 

Physical education is an educational subject that also benefits greatly from the versatility of AR, since it allows students to improve their performance, taking into account its different dimensions: it facilitates understanding of the theoretical part of its contents [53], accelerates the development of complex motor skills [54,55] and improves students’ spatial orientation and interpersonal skills [56,57]. 

Authors such as those of Reference [58], have demonstrated, with the use of AR through the game Pokémon GO in the Physical Education classroom, the increase that is produced in the accomplishment of physical activity in a habitual and playful way in the students. In addition, there is an improvement in cognitive performance, such as memory, attention, concentration, mathematical calculation and linguistic competence, plus a series of benefits that affect emotional intelligence, such as well-being, self-control and sociability [59].

It is considered that AR can be a great weapon against sedentary and obese children if used to create a new learning environment combined with physical training and cognitive learning that encompasses all categories of physical education knowledge [60]. 

If we add to the above benefits the contribution to the development of digital competence, which is so important for the development of the current society [61], the high value of AR as a pedagogical tool in the area of physical education becomes evident [62].

### 1.3. Study Dimensions

This study starts from the analysis of different dimensions, such as socio-educational, motivation, interactions, autonomy, collaboration, deepening of contents, problem solving, class time and grades. The analysis of the scientific literature on how the use of augmented reality impacts on the above dimensions, as well as on different topics, contents and educational levels, justifies the selection of these dimensions. Therefore, in order to continue with the line of research proposed by previous studies, we have considered it appropriate to carry out an analysis of the different dimensions used by different professionals with respect to the subject in question. 

In order to facilitate understanding of the results obtained in this study, we have proceeded to define them separately [63,64,65,66]:

- Socio-educational: It relates variables of a social scope such as age, gender, nationality, place of residence, and of an educational scope, such as the course, methodology, learning difficulties and technologies used.
- Motivation: Indicates the degree of interest shown by students during their teaching and learning process.
- Interactions: Reveals the different types of interaction that can be carried out during the performance of training actions, such as the interaction between teacher and students, between students themselves or between students and the content of the subject.
- Autonomy: It shows what capacities each student has during the implementation of the teaching contents, as well as the degree of autonomy acquired during the development of the teaching.
- Collaboration: Indicates the degree of joint work that the students have developed during the teaching experience.
- Deepening of the contents: It refers to the degree of dedication of the teaching staff with respect to the contents of the subject according to the methodology selected during the teaching practice.
- Problem solving: It indicates the students’ capacity to solve the different difficulties raised by the teacher during the implementation of the didactic contents.
- Class time: Period of time in which the student must acquire the learning contents proposed by the teacher during the course of the class.
- Ratings: These are the self-evaluations developed by the students on the learning acquired during the development of the teaching and learning process.
- Teachers’ ratings: It shows the students’ grades, through the realization of several techniques and evaluation instruments applied by the teachers of the subject.

## 2. Justification and Objectives

Today, the social context profoundly determines both education and the processes that constitute it, mostly because of the omnipresent presence of ICT and the means that develop them. In the teaching reality, this situation is reflected in the development of methodological innovation in which both resources and competencies are imposed for the adequate use of these in the educational context [4,5,6,7].

In this research, an experiment has been carried out which has involved a methodological contrast between different groups. In order to create a didactic unit on spatial orientation using the high school curriculum, we have used, on the one hand, through the application Aurasma, the augmented reality, and on the other hand, we have used a traditional methodology. 

With training based on innovation, the use and inclusion of ICT in educational contexts is promoted, which makes it possible to establish in practice the new principles of education of the present millennium, placing the student as the axis of the didactic process, around which the different ICT resources are implemented with the continuous technological advances [10,11,12,13].

This work is presented as a complement to previous works published in recent times that have shown the effectiveness of augmented reality in education and the processes that comprise it [23,24,25,26,27].

The main objective of this work is to analyse the impact of training action through the use of augmented reality in physical education for the development and acquisition of spatial orientation, as opposed to more traditional training based on the exhibition method. As a result of this general objective, the following research questions (RQ) are articulated:

- RQ1: Do the means used by teachers to monitor tasks influence student motivation?
- RQ2: Do the means used by the teaching staff to follow up the tasks influence the interaction between students and teachers?
- RQ3: Do the means used by teachers to monitor tasks influence students’ interaction with the teaching content?
- RQ4: Do the means used by teachers to monitor tasks influence the interaction between students?
- RQ5: Do the means used by teachers to monitor tasks influence student autonomy?
- RQ6: Do the means used by teachers to monitor tasks influence student collaboration?
- RQ7: Do the resources used by teachers to carry out the tasks influence the level of deepening the content?
- RQ8: Do the means used by the teaching staff to follow up on the tasks influence the degree of resolution of the students’ problems?
- RQ9: Do the means used by the teacher influence the monitoring of class-time tasks by the students?
- RQ10: Does the medium used by teachers to track assignments influence students’ ratings?

## 3. Materials and Methods 

### 3.1. Research Design and Data Analysis

The research is proposed to be quantitative, through a quasi-experimental post-test design which has a descriptive and correlational background, based on the theses of renowned experts [67] and research that has followed this process [68].

An experimental group has been proposed for the present investigation and a control group has also been proposed for the development of the experiment. A different methodology was applied to each group. On the one hand, the control group has received traditional training, that is, an expository methodology without the use of any technological resources. The experimental group has been subjected to a more innovative methodology, since the training process has been applied to it through the use of augmented reality. Thus, the independent variable is defined as the methodological approach used, the dependent variable being the effectiveness achieved in the different dimensions determined. No pre-testing was done on any of the established groups. With the Student’s *t*-test, the aim was to find out if there were significant differences in each of the dimensions, both in the control group and in the experimental group. That is to say, to know if the method applied in one group and another have influenced each of the study dimensions.

The Statistical Package for the Social Sciences (SPSS) v25 program (IBM Corp., Armonk, NY, USA) was chosen to carry out the statistical analysis of the research. In this analysis, basic statistics such as mean (M) and standard deviation (SD) have been developed. The skewness (S_kw_) and kurtosis (K_me_) tests have determined the distribution trend. With the Student’s *t*-test (t_n1+n2-2_), the comparative study of the means between groups has been carried out. Finally, with Cohen’s d and the biserial correlation (r_xy_), the size of the effect achieved has been defined, while taking *p* < 0.05 as a statistically significant difference.

### 3.2. Participants

With a total of 140 students in the first year of the Higher Education, in the subject of Physical Education, a quasi-experimental approach has been proposed. According to relevant and impacting research drawn from the specialized literature, sample size being important is not a determining factor for conducting educational experiments [68,69]. 

Of the students that make up the sample in this study, 58.33% were boys and the rest were girls, with a mean age of 16.8 years (SD = 0.682). This sample was divided into four study groups since the educational centre has four lines or groups/classes of students for the first level of high school. Of these four groups, two were placed as control groups and the other two were placed as experimental groups. The assignment of control and random groups was determined by a random process. (Table 1). 

The sample has been generated for convenience, because of the ease of access to the different subjects. This sample generation technique has been applied in an educational centre in the south of Spain. With respect to attrition bias, in this case, it does not exist because no important data on research participants were lost.

### 3.3. Instrument

An ad hoc questionnaire was designed to carry out the research with reference to previous studies [64,65,66,70,71]. This instrument is structured in thirty-five items distributed in nine dimensions (Socioeducational, Motivation, Interactions, Autonomy, Collaboration, Deepening of contents, Problem solving, Class time and Ratings). The items are exposed through a four-level Likert scale (from 1 = None to 4 = Completely). In addition, teachers’ qualifications have been taken into account.

The Delphi method has been used to diagnose the validity of content, to determine from a qualitative perspective, the vision of multiple doctors and teachers specialised in Physical Education and in the use of information and communication technologies in education (n = 8), which is presented as a positive opinion (M = 4.69; SD = 0.41; min = 1; max = 6) and offered recommendations to optimise the instrument. The experts’ indications focus on the unification of items that are in the same category in order to reduce the total number of items, and on the modification of terms to improve the understanding of the items. This technical feedback was done with the intention of not generating an instrument with a large number of items that would lead to low participation or miscompletion due to lack of interest. The vocabulary was also adjusted to the level of the educational stage so as not to generate biases due to reading comprehension of the questionnaire questions. 

Two statistical procedures such as Kappa by Fleiss and W by Kendall were applied to obtain the degree of agreement and relevance of the judgements made by the specialists to complement this first validation. In these tests, significant values were reached (K = 0.89; W = 0.87) that give prominent relevance to the experts’ view of this validation.

As a result of the exploratory factor analysis established by a varimax rotation and the principal components method, the validation of the construct was optimised. The dependence between the different variables established in the research has been defined by the Bartlett’s test of sphericity (2714.94; *p* < 0.001) and by the Kaiser–Meyer–Olkin (KMO) test, which has established some positive values, and which denote the good sample determination (KMO = 0.88) generated for the said research.

Finally, in order to determine the reliability of the instrument, statistics such as Cronbach’s alpha (α) (0.89) and McDonald’s omega method (0.88), compound reliability (0.83) and Mean variance extracted (0.81) were used. The scores obtained confirm that the instrument has a significant internal consistency suitable for research.

### 3.4. Procedure

The phases that make up the research that is presented here were different. In phase one, the sample was determined, and the selection was made. Following the code of ethics, specifically the code of ethics of the Declaration of Helsinki, we proceeded with a meeting with the school institution for experimentation and access to the sample. The educational institution within the social commitment accepted the execution of the research, which helped both to select the sample and to form and complete the instrument with the consequent informed consent of the participants.

Afterwards, the contents were timed in close collaboration with the members of the Physical Education and Sports Department participating in this work. This educational experience was developed in two 55 min sessions. The month in which it took place was September, in the first didactic unit. The researchers were present at all times in the development of the educational experience, observing the procedure, but never participating in the development. The main content developed in the two sessions was specifically the spatial one, although other contents were also developed (Table 2). 

Next, the areas to carry out the spatial orientation and the orientation points were determined, taking into account that these areas must be characterised by being quite extensive. The selection of points in the area, a total of 15, was established by the teachers who are experts in the subject of physical education and spatial orientation.

In phase two, the Aurasma program was configured. The first thing that was done is the composition of the activating images, and for that, the different orientation points determined in the previous phase were photographed, forming, with these images, the activating image, also called trigger, and then it was geolocated. Once this was done, the program started to produce the virtual layers of augmented reality, in order to give a guide to the student body that pursues the different points of orientation previously determined.

Finally, during this time, the questionnaire was generated and validated, and for its application, a Google Drive form was used. Two sessions were held for each of the participating groups. In session one, a small training on the Aurasma program was given, on its operation and on its configuration for its use on the students’ mobile phones. In the same way, subgroups of 2 people were created, having as criteria the gender and that at least one of the members of the couple had a smartphone. In the second session, the activity was started in the determined area in such a way that the couples took the exit every three minutes. In the case of the couples who finished the activity early, they could support those who were having more difficulty. The activity ended with a time of feedback, reflection and evaluation of the activity, before filling in the questionnaires through a link provided to the students. 

## 4. Results

The data presented after applying the descriptive statistical study of the students who are in high school show, in general terms, that there are very disparate mean differences between the control group and the experimental group. In the control group, the dimensions with the highest means were teacher ratings and ratings. In contrast, in the experimental group, the motivation dimension was the most important. The measures in the control group are 2 points, except for ratings and teacher ratings, which exceeded this barrier, but with a very slight difference. In the experimental group, all dimensions exceeded 3 points. This shows a difference of one point between the dimensions of the control group and the experimental group. The distribution of the values of all dimensions is considered normal, since the statistics of asymmetry and kurtosis were in the range of +1.96 and −1.96 [72]. According to the values of the standard deviation, in the control group, the answers were shown grouped in all the dimensions, except in student-content. In the experimental group, the answers were more dispersed, except in the dimensions motivation, deepening and teacher-ratings. The kurtosis presented in all dimensions, both in the control group and in the experimental group, is platicuric (Table 3). 

**Figure 1:**
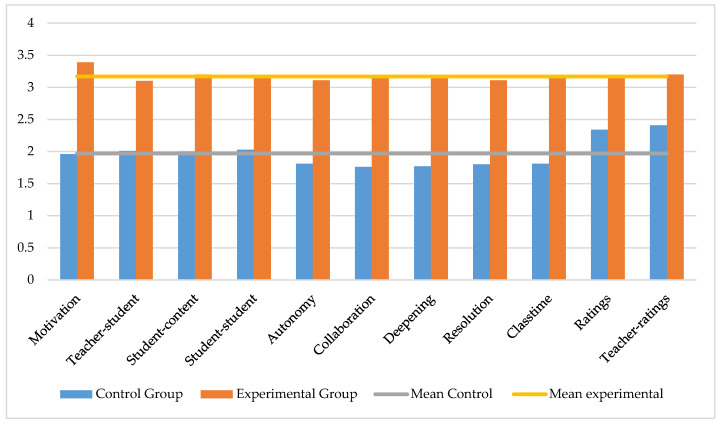
Comparison between control group and experimental group.

The comparison of means, represented in Figure 1, shows that the responses in all the dimensions of the experimental group were even, except in ratings and teacher ratings, where they exceeded the total mean. In the experimental group, something similar occurred. The response pattern in all dimensions was even, except in motivation, where it exceeded the total mean. This determines that the teaching methods generated a similar effect in all the study dimensions.

The degree of independence of the results obtained in the teaching and learning method developed in the control group, in which a traditional teaching method has been applied, and the pedagogical process of the experimental group, which has developed a pedagogical process using augmented reality, have been identified thanks to the Student’s t statistic for independent samples. The data show a significant relationship, to a very high degree, between all the dimensions of studies. Mean differences were greater than one point in all dimensions, except in ratings and teacher ratings, where it was less than one point. In all cases, the differences in means were in favour of the experimental group. The greatest difference in means occurred in the motivation dimension. The strength of association of the dimensions was medium–high. The effect size, marked by Cohen’s d, shows that the effect size was very low, except in the collaboration, deepening, resolution and class-time dimensions, where the effect size was low (Table 4).

## 5. Discussion

The incorporation of technological resources in classrooms is becoming an increasingly common practice within Secondary Education classrooms. Specifically, in the area of physical education, more and more teachers are betting on these emerging tools when carrying out their teaching practice, due to the amount of benefits they contribute to students [3].

As a result of this idea, the present study aimed to analyse from a quasi-experimental perspective how the application of AR affected multiple attitudinal and skill variables in students in the first year of high school. The results extracted in the statistical analyses after the experience was carried out allowed us to observe how the experimentation with AR improved the totality of the variables evaluated, most of which obtained homogeneous results. Specifically, the constructs related to motivation, in-depth study of the content of the subject and teacher-training were those that obtained the highest scores. Therefore, we continue on a path of research that consolidates the idea of the motivational improvement caused by experimentation with technological resources in the classroom, and more specifically, with AR [25,28]. Similarly, making contact with this resource made students improve their perception of the subject of physical education, and led to an interest in deepening their knowledge, which in this case was about spatial orientation. Thus, AR has been elucidated as a resource that has stimulated students to go deeper into the subject, due to its dynamic and empirical character, which is in opposition to traditional expository teaching [37]. 

On the other hand, concerning skill variables, the analysis detected, as skills referring to autonomy, problem solving or academic performance, which promoted a growth in contrast to the control group. A line was established that coincides with previous studies that consolidate the idea about the methodological change caused by the implementation of this type of resource, where the role of the student turns towards discovery and action [31].

In contrast, the results obtained by the control group denoted standard results in practically all of the variables analysed. This denotes the feeling of obsolescence that purely expository teaching holds, which reduces the student to a passive and uninteresting role [7]. In this sense, the application of the t-test allowed us to corroborate the difference between the means obtained in the different treatment groups. This allowed us to validate the existing differences between the treatment groups involved in the research.

Finally, and based on the ten research questions posed at the beginning of this manuscript, the analysis of results has provided answers to each of them. In this case, in each of them, a positive resolution has been given in favour of the application of AR as a classroom resource. Each and every one of the study variables experienced an improvement after the experimentation with this resource.

## 6. Conclusions

The irruption of information and communication technologies is a palpable reality in the education system. They offer a wide range of resources that provide multiple possibilities for innovative and quality education. Faced with this situation, teachers are faced with the challenge of training in their operation, as well as knowing the many learning opportunities they provide when it comes to transmitting a teaching opportunity.

Around this paradigm, the present study extracted interesting data on the effects caused in the students of the first year of High School around the application of AR in the subject of physical education. The statistical analyses used after the experimentation with AR for students promoted an improvement in motivation, academic performance or receptivity towards the area of knowledge among the students. Therefore, this is a resource that is presented as an opportunity to potentiate teaching in the area of physical education.

As for the limitations of the study, there is the low level of effect obtained by the comparative results between the control and experimental groups. This is due to the sample size, which was not high and was selected through convenience sampling. However, it allows us to establish an approach to the idea of how the application of AR may affect different variables in the study. On the other hand, as future lines of research, the need for experts to continue contributing empirical experiences in which the effectiveness of different emerging resources, such as AR in the area of physical education as well as in the rest of the areas of knowledge, can be verified, is advocated. It is recommended that professionals in the different educational stages promote an educational praxis through ICT, which allows the creation of sustainable learning ecosystems and the promotion of active learning in the student body.

In conclusion, the application of technological resources was presented as a real alternative to put into practice in the area of physical education. They are tools that provide a new vision when it comes to accessing knowledge, and that allow us to dynamize and better attend to the diversity that exists in the classroom. In this way, the aim is to establish a change from an obsolete traditional teaching to a different way of building learning, which is inherent to the time frame and the student body of today.

## Figures and Tables

**Table 1:** Research design.

Group	n	Composition	Pre-Test	Treatment	Post-Test
1: Control	30	Natural	-	-	O_1_
2: Experimental	30	Natural	-	X	O_2_
3: Control	30	Natural	-	-	O_3_
4: Experimental	30	Natural	-	X	O_4_

Note: Treatment was randomly assigned.

**Table 2:** Contents worked on during the sessions in the control group and in the experimental group.

Control Group	Experimental Group
Deepening the work of physical conditioning focused on one’s own needs.	Deepening the work of physical conditioning focused on one’s own needs.
Specific activities of activation and recovery of efforts.	Specific activities of activation and recovery of efforts.
Awareness of one’s own characteristics and possibilities.	Awareness of one’s own characteristics and possibilities.
The norm as an element regulating behaviour in social-motor activities.	The norm as an element regulating behaviour in social-motor activities.

**Table 3:** Results obtained for the dimensions of study in the control group and experimental group of Higher Education.

Group		Likert Scale *n (%)*				Parameters			
	Dimensions	None	Few	Enough	Completely	M	SD	S_kw_	K_me_
**Control Group**	Motivation	27(38.6)	23(32.9)	16(22.9)	4(5.7)	1.96	0.924	0.541	−0.719
	Teacher-student	26(37.1)	22(31.4)	17(24.3)	5(7.1)	2.01	0.955	0.484	−0.833
	Student-content	29(41.4)	18(25.7)	17(24.3)	6(8.6)	2.00	1.01	0.526	−0.954
	Student-student	27(38.6)	20(28.6)	17(24.3)	6(8.6)	2.03	0.992	0.491	−0.918
	Autonomy	35(50)	17(24.3)	14(20)	4(5.7)	1.81	0.952	0.800	−0.553
	Collaboration	37(52.9)	15(21.4)	16(22.9)	2(2.9)	1.76	0.908	0.746	−0.758
	Deepening	37(52.9)	15(21.4)	15(21.4)	3(4.3)	1.77	0.935	0.807	−0.627
	Resolution	36(51.4)	16(22.9)	14(20)	4(5.7)	1.80	0.957	0.825	−0.544
	Class-time	37(52.9)	12(17.1)	18(25.7)	3(4.3)	1.81	0.967	0.683	−0.964
	Ratings ^a^	16(22.9)	22(31.4)	24(34.3)	8(11.4)	2.34	0.961	0.060	−0.967
	Teacher-ratings ^a^	14(20)	24(34.3)	21(30)	11(15.7)	2.41	0.985	0.106	−0.975
**Experimental Group**	Motivation	1(1.4)	14(20)	12(17.1)	43(61.4)	3.39	0.856	−0.988	−0.476
	Teacher-student	10(14.3)	12(17.1)	9(12.9)	39(55.7)	3.10	1.14	−0.799	−0.941
	Student-content	8(11.4)	12(17.1)	8(11.4)	42(60)	3.20	1.09	−0.952	−0.639
	Student-student	9(12.9)	9(12.9)	14(20)	38(54.3)	3.16	1.08	−0.952	−0.512
	Autonomy	10(14.3)	10(14.3)	12(17.1)	38(54.3)	3.11	1.12	−0.862	−0.767
	Collaboration	9(12.9)	10(14.3)	13(18.6)	38(54.3)	3.14	1.09	−0.907	−0.625
	Deepening	6(8.6)	11(15.7)	19(27.1)	34(48.6)	3.16	0.987	−0.883	−0.361
	Resolution	7(10)	12(17.1)	17(24.3)	34(48.6)	3.11	1.02	−0.810	−0.608
	Class-time	6(8.6)	13(18.6)	16(22.9)	35(50)	3.14	1.01	−0.814	−0.602
	Ratings ^a^	7(10)	11(15.7)	14(20)	38(54.3)	3.19	1.04	−0.943	−0.453
	Teacher-ratings ^a^	5(7.1)	13(18.6)	15(21.4)	37(52.9)	3.20	0.987	−0.883	−0.480

^a^ Established grade group (None: 1–4.9; Few: 5–5.9; Enough: 6–8.9; Completely: 9–10).

**Table 4:** Study of the value of independence between the control group and experimental group.

Dimensions	µ (X_1_–X_2_)	*t_n1+n2-2_*	df	*d*	r_xy_
Motivation	−1.429(1.96–3.39)	−9.490 **	138	0.079	0.628
Teacher-student	−1.086(2.01–3.10)	−6.095 **	138	0.029	0.461
Student-content	−1.200(2.00–3.20)	−6.738 **	138	0.070	0.498
Student-student	−1.129(2.03–3.16)	−6.421 **	138	0.033	0.480
Autonomy	−1.300(1.81–3.11)	−7.385 **	138	0.087	0.532
Collaboration	−1.386(1.76–3.14)	−8.155 **	138	0.110	0.570
Deepening	−1.386(1.77–3.16)	−8.525 **	138	0.128	0.587
Resolution	−1.314(1.80–3.11)	−7.824 **	138	0.120	0.554
Class-time	−1.329(1.81–3.14)	−7.942 **	138	0.151	0.560
Ratings ^a^	−0.843(2.34–3.19)	−4.980 **	138	−0.009	0.390
Teacher-ratings ^a^	−0.786(2.41–3.20)	−4.714 **	138	−0.009	0.372

** The correlation is significant at the 0.01 level; ^a^. Established grade group (None: 1–4.9; Few: 5–5.9; Enough: 6–8.9; Completely: 9–10); Note: µ: Mean differences; *t_n1+n2-2_*: Student’s t-test; df: Degrees of freedom; d: Cohen’s d; r_xy_: Biserial Correlation.
